# Paediatric Acute Respiratory Distress Syndrome Neuromuscular Blockade study (PAN-study): a phase IV randomised controlled trial of early neuromuscular blockade in moderate-to-severe paediatric acute respiratory distress syndrome

**DOI:** 10.1186/s13063-021-05927-w

**Published:** 2022-01-31

**Authors:** Michelle W. Rudolph, Sjoerdtje Slager, Johannes G. M. Burgerhof, Job B.M. van Woensel, Jan-Willem C. Alffenaar, Roelie M. Wösten - van Asperen, Matthijs de Hoog, Marloes M. IJland, Martin C. J. Kneyber, For the SKIC research consortium

**Affiliations:** 1 Department of Paediatrics, Division of Paediatric Critical Care Medicine, Beatrix Children’s Hospital, University Medical Centre Groningen, University of Groningen, Groningen, The Netherlands; 2 Department of Epidemiology, University Medical Centre Groningen, University of Groningen, Groningen, The Netherlands; 3 Department of Paediatric Intensive Care, Emma Children’s Hospital/Amsterdam University Medical Centre, Amsterdam, The Netherlands; 4 Department of Clinical Pharmacy and Pharmacology, University Medical Centre Groningen, University of Groningen, Groningen, The Netherlands; 5 Faculty of Medicine and Health, School of Pharmacy, University of Sydney, Sydney, NSW Australia; 6 Westmead Hospital, Sydney, NSW Australia; 7 Marie Bashir Institute of Infectious Diseases and Biosecurity, University of Sydney, Sydney, Australia; 8 Paediatric Intensive Care Unit, Wilhelmina Children’s Hospital/University Medical Centre Utrecht, Utrecht, The Netherlands; 9 Intensive Care Unit, Departments of Paediatrics and Paediatric Surgery, Erasmus MC-Sophia Children’s Hospital, Rotterdam, The Netherlands; 10 Department of Paediatric Intensive Care, Radboud University Nijmegen Medical Centre, Nijmegen, The Netherlands; 11 Critical care, Anaesthesiology, Peri-operative & Emergency medicine (CAPE), University Medical Centre Groningen, University of Groningen, Groningen, The Netherlands

**Keywords:** Acute respiratory distress syndrome, Mechanical ventilation, Children, Neuromuscular blockade, Respiratory morbidity, Critical illness polyneuropathy and myopathy, Respiratory morbidity score

## Abstract

**Background:**

Paediatric acute respiratory distress syndrome (PARDS) is a manifestation of severe, life-threatening lung injury necessitating mechanical ventilation with mortality rates ranging up to 40–50%. Neuromuscular blockade agents (NMBAs) may be considered to prevent patient self-inflicted lung injury in PARDS patients, but two trials in adults with severe ARDS yielded conflicting results. To date, randomised controlled trials (RCT) examining the effectiveness and efficacy of NMBAs for PARDS are lacking. We hypothesise that using NMBAs for 48 h in paediatric patients younger than 5 years of age with early moderate-to-severe PARDS will lead to at least a 20% reduction in cumulative respiratory morbidity score 12 months after discharge from the paediatric intensive care unit (PICU).

**Methods:**

This is a phase IV, multicentre, randomised, double-blind, placebo-controlled trial performed in level-3 PICUs in the Netherlands. Eligible for inclusion are children younger than 5 years of age requiring invasive mechanical ventilation with positive end-expiratory pressure (PEEP) ≥ 5 cm H_2_O for moderate-to-severe PARDS occurring within the first 96 h of PICU admission. Patients are randomised to continuous infusion of rocuronium bromide or placebo for 48 h. The primary endpoint is the cumulative respiratory morbidity score 12 months after PICU discharge, adjusted for confounding by age, gestational age, family history of asthma and/or allergy, season in which questionnaire was filled out, day-care and parental smoking. Secondary outcomes include respiratory mechanics, oxygenation and ventilation metrics, pulmonary and systemic inflammation markers, prevalence of critical illness polyneuropathy and myopathy and metrics for patient outcome including ventilator free days at day 28, length of PICU and hospital stay, and mortality

**Discussion:**

This is the first paediatric trial evaluating the effects of muscular paralysis in moderate-to-severe PARDS. The proposed study addresses a huge research gap identified by the Paediatric Acute Lung Injury Consensus Collaborative by evaluating practical needs regarding the treatment of PARDS. Paediatric critical care practitioners are inclined to use interventions such as NMBAs in the most critically ill. This liberal use must be weighed against potential side effects. The proposed study will provide much needed scientific support in the decision-making to start NMBAs in moderate-to-severe PARDS.

**Trial registration:**

ClinicalTrials.govNCT02902055. Registered on September 15, 2016.

## Administrative information

**Table:** 

Title {1}	Paediatric ARDS Neuromuscular blockade study (PAN-study): A phase IV randomised controlled trial of early neuromuscular blockade in moderate-to-severe paediatric acute respiratory distress syndrome
Trial registration {2a and 2b}.	ClinicalTrials.gov NCT02902055, September 15, 2016
Protocol version {3}	Version 7; March 1, 2019
Funding {4}	Grant from ZonMw, number: 848041002
Author details {5a}	(1) Department of Paediatrics, Division of Paediatric Critical Care Medicine, Beatrix Children’s Hospital, University Medical Centre Groningen, University of Groningen, Groningen, the Netherlands; (2) Department of Epidemiology, University Medical Centre Groningen, University of Groningen, Groningen, the Netherlands; (3) Department of Paediatric Intensive Care, Emma Children's Hospital/Amsterdam University Medical Centre, Amsterdam, The Netherlands; (4) Department of Clinical Pharmacy and Pharmacology, University Medical Centre Groningen, University of Groningen; (5) University of Sydney, Faculty of Medicine and Health, School of Pharmacy, Sydney, NSW, Australia; (6) Westmead Hospital, Sydney, NSW, Australia; (7) Marie Bashir Institute of Infectious Diseases and Biosecurity, University of Sydney, Sydney, Australia; (8) Paediatric Intensive Care Unit, Wilhelmina Children's Hospital/University Medical Centre Utrecht, Utrecht, The Netherlands; (9) Intensive Care Unit, Departments of Paediatrics and Paediatric Surgery, Erasmus MC-Sophia Children's Hospital; (10) Department of Paediatric Intensive Care, Radboud University Nijmegen Medical Centre, Nijmegen, the Netherlands; (11) Critical care, Anaesthesiology, Peri-operative & Emergency medicine (CAPE), University Medical Centre Groningen, University of Groningen, Groningen, the Netherlands
Name and contact information for the trial sponsor {5b}	Marjo Tieleman; Tieleman@zonmw.nl
Role of sponsor {5c}	The sponsor has no role in the study design, collection, management, analysis nor in the interpretation or publication of the data.

## Background and rationale {6a}

Paediatric acute respiratory distress syndrome (PARDS) is a manifestation of severe, life-threatening lung injury. The prevalence of PARDS in critically ill, mechanically ventilated children may be as high as 10% of all children admitted to the paediatric intensive care unit (PICU) with mortality rates ranging up to 40–50% [1]. The disease is characterised by massive pulmonary inflammation, alterations in surfactant homeostasis and ventilation/perfusion mismatching leading to severe hypoxemia and multiple organ dysfunction [2]. Mechanical ventilation (MV) has added significantly to the survival of PARDS patients but also induces a pulmonary inflammation (biotrauma) that aggravates pre-existing lung injury (double-hit), a concept known as ventilator-induced lung injury (VILI) [3–5].

Critical care practitioners have adopted the philosophy of maintaining spontaneous breathing in mechanically ventilated patients as much as possible. However, a study in adults with severe ARDS challenged these practices. Early use of neuromuscular blocking agents (NMBAs) in adults with severe ARDS, defined as PaO_2_/FiO_2_ ratio less than 150 mmHg, resulted in improved 90-day survival and increased time off the ventilator without increasing muscle weakness [6]. In this study, 340 patients with early severe ARDS, meeting criteria within 48 h of ICU admission, were randomised to cisatracurium besylate or placebo once adequately sedated. After adjustment for baseline PaO_2_/FiO_2_, plateau pressure and the Simplified Acute Physiology Score, the cisatracurium group had a hazard ratio for death at 90 days of 0.68, (95% confidence interval [CI], 0.48 to 0.98; *P* = 0.04), compared to the placebo group. These findings remained consistent when combined in a meta-analysis with earlier, smaller studies from the same group of investigators [7]. Additional beneficial effects of NMBAs observed included sustained improvement in oxygenation, less organ dysfunction, and a lower pro-inflammatory response [8–10]. However, the practice change that followed this trial came under scrutiny after the publication of the Re-evaluation Of Systemic Early Neuromuscular Blockade (ROSE) trial in 2019 [11]. This trial was designed to determine the safety and efficacy of early NMBAs with concomitant heavy sedation as compared with a strategy of usual care with lighter sedation targets. It was prematurely terminated for futility after the inclusion of 1006 patients because no difference in 90-day survival (42·5% vs 42·8%) was found.

To date, randomised controlled trials (RCT) examining the effectiveness and efficacy of NMBAs for paediatric ARDS are lacking. Our group found that continuous administration of NMBAs significantly improved oxygenation in patients with moderate-to-severe PARDS [12]. The Paediatric Acute Lung Injury Consensus Conference (PALICC) and the Paediatric European Mechanical Ventilation Consensus Conference (PEMVECC) recommended that clinical trials investigating the short- and long-term outcomes of NMBA use are much needed, especially as—despite the lack of evidence—NMBAs are often used in the most critically ill paediatric patients [13–18]. Furthermore, the possible beneficial effects of NMBAs must be outweighed against side-effects such as critical illness polyneuropathy and myopathy (CIPNM), a phenomenon that has been observed especially in adults who are on concurrent corticosteroids or have renal failure [19]. Limited data suggest the prevalence of CIPNM in children is very low [20].

### Objectives {7}

The primary objective is to test the hypothesis that the use of NMBAs for 48 h in paediatric patients younger than 5 years of age with early moderate-to-severe PARDS will lead to at least a 20% reduction in cumulative respiratory morbidity score 12 months after discharge from the PICU.

The secondary objectives are to evaluate the effects of NMBAs on pulmonary and systemic inflammation, metrics for oxygenation and ventilation, non-respiratory organ dysfunction, and respiratory system mechanics.

Exploratory objectives: ventilator free days (VFD) at day 28, PICU and hospital length of stay, 90-day mortality, concomitant use of sedatives, complications (e.g. adverse drug reactions, reintubation rate, critical illness polyneuropathy and myopathy, ventilator-associated pneumonia (VAP), withdrawal syndrome, delirium), the course of the respiratory morbidity score at 3, 6 and 9 months and lung function (lung clearance index (LCI), functional residual capacity (FRC).

### Trial design {8}

The Paediatric ARDS Neuromuscular blockade (PAN) trial is a phase IV, multicentre, randomised, double-blind, placebo-controlled superiority trial.

## Methods: Participants, interventions and outcomes

### Study setting {9}

The study will be conducted at five level-3 PICUs in the Netherlands: UMC Groningen (Groningen), UMC Utrecht (Utrecht), Amsterdam UMC (Amsterdam), ErasmusMC (Rotterdam) and RadboudUMC (Nijmegen).

### Eligibility criteria {10}

Eligible for inclusion are children younger than 5 years of age (< 20kg) with an indwelling arterial/venous catheter in situ, requiring invasive MV with positive end-expiratory pressure (PEEP) ≥ 5 cm H_2_O for moderate-to-severe PARDS occurring within the first 96 h of PICU admission. Moderate-to-severe PARDS is defined by acute onset of disease, oxygenation index (OI) > 12/oxygen saturation index (OSI) > 9.9, one or more infiltrates on chest radiograph and no evidence of left ventricular failure or fluid overload.

Exclusion criteria are:

- Known allergy or intolerance to rocuronium
- Continuous administration of neuromuscular blockade prior to meeting PARDS criteria/start of study
- Bolus administration of neuromuscular blockade within 1 h before meeting PARDS criteria/start of study
- Chronic respiratory failure on home ventilation
- Intracranial hypertension
- Pre-existing pulmonary hypertension
- Congenital heart disease with left-to-right shunting
- (Suspected) underlying neuromuscular or metabolic disorders
- Bone marrow transplantation
- Expected duration of MV less than 48 h
- Withdrawal of life-sustaining treatment or other treatment limitations

### Who will take informed consent? {26a}

Site investigators will screen for eligible subjects daily. Screening logs will be used to facilitate the screening process and provide an auditable record of potentially eligible subjects. Parents or legal caretakers are informed by a member of the study team about the study as soon as possible when a patient becomes eligible for inclusion. The study team will explain the study to the parents and ask for informed consent. Parents/legal caretakers will have 12 h to consider giving consent, provided that this period does not exceed the 96-h interval between time of PICU admission and study enrolment. At least one parent/legal caretaker must give written consent while the other can give verbal consent to include the subject, hereafter written consent will be gained within 48 h after inclusion.

### Additional consent provisions for collection and use of participant data and biological specimens in ancillary studies {26b}

Participant data and biological specimens will not be used in ancillary studies.

## Interventions

### Explanation for the choice of comparators {6b}

A comparator was chosen as the control in this trial in order to ensure blinding by having similar quantities of fluids administered in the intervention and control arm. Isotonic saline was chosen as the placebo because this is a commonly used infusion fluid in the PICU. Patients in the control arm receive an isotonic saline bolus of 0.1 mL/kg followed by a continuous infusion of 0.1 mL/kg/h isotonic saline for 48 h.

### Intervention description {11a}

Rocuronium is a non-depolarizing neuromuscular blocker widely used to produce muscle relaxation to help facilitate surgery and ventilation of the lungs in elective and emergent situations. It is one of the many non-depolarizing neuromuscular blockers used but has the distinct advantage of being fast-acting and reversible [21]. It is often used in critically ill, mechanically ventilated children when sedation alone is inadequate to achieve effective mechanical ventilation [22]. The occurrence of allergic or anaphylactic reactions is a known risk for rocuronium; its occurrence in children appears very rare as to date only three case reports have been published [23]. Muscular weakness following prolonged infusion of rocuronium is another risk; there is very little data on the occurrence of ICU acquired weakness in children. One group of investigators found no association between ICU acquired weakness and neuromuscular blocking agents when retrospectively analysing data from 203.875 PICU admissions in the period 2009–2013 (with 55 cases of ICU acquired weakness—i.e. 0.03%) [24].

Patients in the intervention arm will receive 0.1 mL/kg bolus of rocuronium bromide 10 mg/mL (compatible with the recommended dosage of 1 mg/kg) followed by a continuous infusion of rocuronium bromide 10 mg/mL at a rate of 0.1 mL/kg/h (compatible with the recommended dosage of 1 mg/kg/h) (investigational product) for 48 h.

### Criteria for discontinuing or modifying allocated interventions {11b}

An investigator may discontinue or withdraw a patient from the study for the following reasons:

- withdrawal of informed consent by the parents or the legal caretakers
- signs of hypersensitivity or an allergic reaction that is not attributable to any concurrent medication, defined by skin rash and/or hypotension and/or severe bronchospasm necessitating the use of anti-histaminic drugs and corticosteroids and/or vasopressors
- cannulation for extra-corporeal life support (ECLS)

### Strategies to improve adherence to interventions {11c}

Most of the interventions take place during admission on the PICU by research staff. The respiratory morbidity score questionnaires will be administered electronically by email and reminders will be sent automatically after 1 week.

### Relevant concomitant care permitted or prohibited during the trial {11d}

Standard of care is provided for all enrolled patients. It is guided by clinical judgement, local guidelines, and international recommendations from PALICC and PEMVECC, and includes ventilator management, sedation and analgesia use, nutrition, transfusion management and hemodynamic and fluid management [17, 25]. Ventilator management goals are adequate oxygenation and ventilation, defined by pulse oximetry oxygen saturation (SpO_2_) 88–92% and 7.20 ≤ pH ≤ 7.35 (irrespective of PaCO_2_) during the acute phase of disease. PALICC recommendations include expiratory tidal volume (Vte) 5–7 ml/kg ideal body weight (IBW), peak inspiratory pressure (PIP) ≤ 28 cm H_2_O (may allow up to 32 cm H_2_O for patients with poor chest wall compliance, i.e. those with increased chest wall stiffness or increased abdominal pressure) and PEEP set by FiO_2_ and respiratory system mechanics. Use of pulmonary specific and non-specific ancillary treatment including inhaled nitric oxide (iNO), steroids, surfactant and prone positioning are also at the discretion of the local bedside team. Use of escalating interventions such as high-frequency oscilattory ventilation (HFOV) or ECLS is also at the discretion of the bedside team. Ventilation treatment failure is defined as a 4-h pattern of either persistent hypoxia (SaO_2_< 85%) with FiO_2_ 1.0 and max PEEP or persistent hypoventilation (pH < 7.20) with PIP > 35 cmH_2_O and a respiratory rate that does not cause intrinsic PEEP. Onset of weaning is at the discretion of the attending physician. Once deemed eligible for weaning, a daily extubation readiness test (ERT) is performed per institutional algorithm.

Patients will be managed using a conservative fluid strategy. The goal is adequate cardiac output to meet the metabolic needs of the patient, specifically, a normal blood pressure for age, brisk capillary refill, and adequate peripheral perfusion to achieve adequate end organ perfusion. The care team will delineate daily mean arterial blood pressure goals. Hemodynamic management may be guided by devices such as pulse contour cardiac output (PiCCO). Diuretic therapy is used to achieve desired fluid balance. Type of drug, starting and maintenance dose is at the discretion of the local care team, taking the goals of fluid management into account. Use of vaso-active medications is also at the discretion of the care team. Maintenance fluids are calculated per standard paediatric practice according to local guidelines. The care team will determine the type of fluid (colloids, crystalloids) administered. All fluids, including IV continuous infusions, IV intermittent medications, blood products, IV and enteral nutrition, will contribute to the patient’s hourly total. Medications should be administered using the least amount of fluid possible.

Patient’s level of comfort is assessed per phase of illness and criticality at least every 4 h while intubated. The Comfort B score is used per discretion of the local care team in all patients, although it must be noted that it cannot be used in paralysed patients [26]. In all patients, patient comfort is also deemed insufficient if there is a persistent > 20% increase in heart rate and blood pressure either spontaneously or with stimulation despite managing obvious causes for discomfort such as (partial) endotracheal tube blockage, mucus retention or constipation. If present, sedation and/or analgesia regimens can be modified according to local guidelines. Centres may substitute drugs within a class (e.g. narcotics - morphine or fentanyl or benzodiazepines - midazolam and lorazepam) and by route of administration (e.g. enteral for intravenous). Alternative measures such as the analgesia nociception index (ANI) require additional monitoring devices that are not available in all participating centres. Local screening tools for withdrawal and/or delirium will be used.

### Provisions for post-trial care {30}

A participant insurance by the UMCG covers all participants who suffer harm from trial participation with a maximum of €650.000 per subject, €5.000.000 max per study and €7.500.000 per year.

### Outcomes {12}

#### Primary outcome measure

Cumulative respiratory morbidity score 12 months after PICU discharge, adjusted for confounding by age, gestational age, family history of asthma and/or allergy, season in which questionnaire was filled out, day-care and parental smoking.

#### Secondary outcome measures

Secondary outcomes include LCI and FRC measured using multiple breath wash-out 12 months after PICU discharge, and the level and time course of the cumulative respiratory morbidity score assessed at baseline, 3, 6 and 9 months after PICU discharge. Secondary outcome measures related to the acute phase of disease (i.e., during PICU admission) include level and time course of ventilator settings (peak inspiratory pressure, plateau pressure, mean airway pressure, driving pressure, PEEP, set rate, total rate, inspiratory time), respiratory system mechanics (quasistatic compliance, dynamic compliance, respiratory system resistance), metrics of oxygenation (SpO_2_/FiO_2_ ratio, PaO_2_/FiO_2_ ratio, OI, OSI) and ventilation (PCO_2_, pH), and hemodynamic parameters (heart rate, blood pressure, central venous pressure, daily cumulative fluid balance, number of fluid challenges). We will measure the level and time course of the non-respiratory pediatric logistic organ dysfunction 2 (PELOD – 2) score as measure of organ dysfunction and pediatric Risk, Injury, Failure, End-stage renal failure (pRIFLE) criteria for acute kidney injury, the number of adverse drug reactions, and re-intubations [27, 28]. We will study the level and time course of the pulmonary and systemic inflammatory response, the prevalence of critical illness polyneuropathy and myopathy, and ventilator-associated pneumonia.

##### Exploratory outcome measures

These include use of (non-) pulmonary ancillary treatment (e.g. steroids, inhaled drugs, prone positioning) and HFOV, daily cumulative dosage sedatives and analgesics, prevalence of drug withdrawal symptoms and delirium, ventilator-free days at day 28 (VFD), length of MV, length of PICU stay and mortality. We will also explore changes in Pediatric Cerebral Performance Category and Pediatric Outcome Performance Category.

### Participant timeline {13}

A schema of this trial and a timeline is presented in the SPIRIT figure below (see Table 1). The total study duration will be the length of PICU admission plus 12 months follow-up. Patients receive the intervention during the first 48 h after enrolment.

**Table 1:**
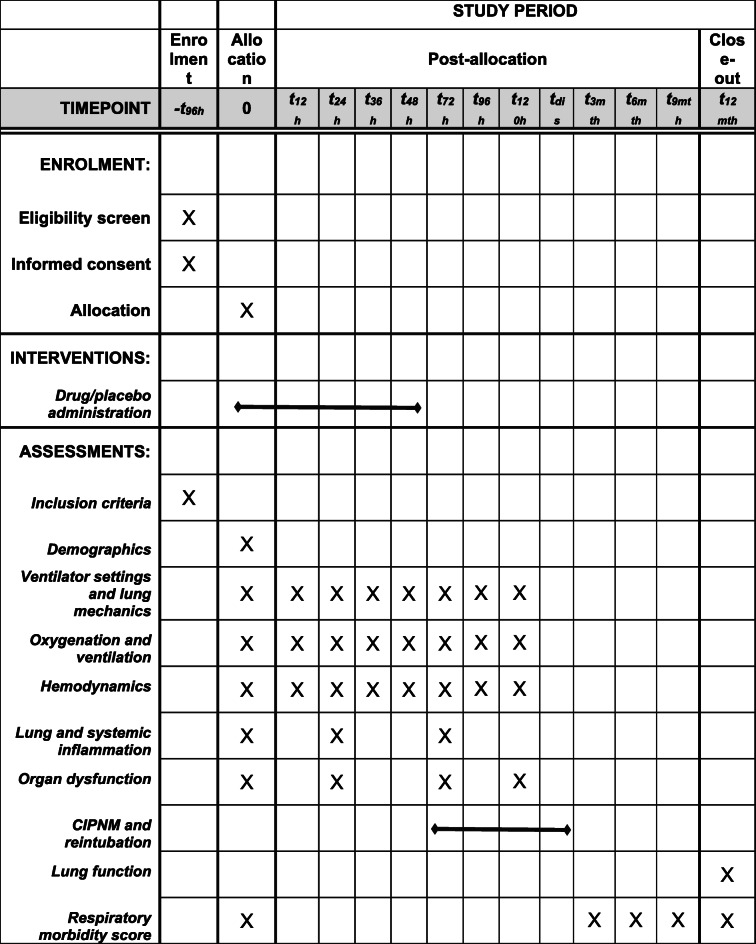
Table 1

### Sample size {14}

The primary outcome is the cumulative respiratory morbidity score 12 months after PICU discharge. A classical sample size based on a independent sample *t* test between two groups was calculated, assuming a 20% difference in cumulative mean respiratory morbidity score. This resulted in a required sample size of 148 patients (*N* = 74 intervention versus *N* = 74 placebo) to demonstrate a 20% reduction in respiratory symptoms in the intervention group compared to the placebo with an alpha of 5% and power of 80%. Considering a potential maximum drop-out of 20% per group because of withdrawal of consent or loss to follow-up, we need to enrol *N* = 89 patients per group. Thus, the total sample size is 178 patients.

### Recruitment {15}

All potential subjects will be informed about the study as soon as possible, even prior to meeting the criteria due to the limited time for enrolment when criteria are met.

## Assignment of interventions: allocation

### Sequence generation {16a}

Patients will be randomised to rocuronium bromide or isotonic saline by the ServiceDesk Clinical Research Office (SD-CRO, UMCG). They will create a randomisation list for the whole study, ahead of time, using blocked randomisation with a block size of 4.

### Concealment mechanism {16b}

The A15 Pharmacy of the UMC Groningen, coordinated by the Department of Clinical Pharmacy and Pharmacology of the UMC Groningen, will prepare and label the study medication. The rocuronium bromide will be refilled under good manufacturing practice (GMP) conditions into a new 10 mL vials to match placebo, that will be adequately labelled for the study. The NaCl 0.9% will be put in a vial matching the rocuronium vial and will also be adequately labelled for the study.

The rocuronium bromide as well as the NaCl 0.9% will be packaged in non-transparent boxes per 10 vials. This way, one box of vials will contain sufficient study medication for one patient for 48 h. The non-transparent boxes will ensure blinding of the study team in case of slight colour differences between rocuronium bromide and the NaCl 0.9%.

### Implementation {16c}

The study team enrols the participant and always uses the first blinded kit (lowest number) available and hereby assigns them (blinded) to the study arm and the interventions of the study.

## Assignment of interventions: Blinding

### Who will be blinded {17a}

Full blinding of the clinical team cannot be ensured because children randomised to the control arm may display spontaneous movements and/or breathing. For this reason, the study team will be prohibited from participating by any means in the clinical care of the participant. On top of that, the statistical analysis and long-term outcome assessment staff are blinded to the study arm, and aggregate outcome data will be restricted to an unblinded statistician and the data safety monitoring board.

### Procedure for unblinding if needed {17b}

The randomisation schedule is provided by the ServiceDesk Clinical Research Office and kept by the Department of Clinical Pharmacy and Pharmacology of the UMC Groningen to ensure blinding until the end of the study. In case of a medical emergency, the pharmacist on call can be consulted for unblinding.

## Data collection and management and storage of biological specimens

### Plans for assessment and collection of outcomes {18a}

#### Primary outcome

The respiratory morbidity will be assessed using a structured questionnaire in agreement with recommendations from the American Thoracic Society [29]. This parental questionnaire studies the presence of respiratory symptoms including cough, wheezing and shortness of breath during rest and/or activity. The questionnaire consists of 14 items that can only be answered by yes or no. The questionnaire is commonly used and validity of the items in the respiratory morbidity score has been shown [29, 30]. Upon discharge, parents or legal caretakers are asked for their e-mail address and phone number; the personalised link to the web-based questionnaire will be e-mailed to them 12 months after PICU discharge. If there is no response within 7 days, they will receive a reminder via e-mail. The questionnaire is personalised and anonymised. The same questionnaire is used upon PICU admission to study baseline respiratory morbidity.

#### Secondary outcomes

Data for the secondary and exploratory outcome measures will primarily be collected during the acute phase of disease, when patients are admitted to the PICU. Ventilator settings and respiratory system parameters will be read from the ventilator. Blood sampling will be done to assess pCO_2_ and pH; to calculate the PaO_2_/FiO_2_ ratio and the OI (calculated by [mean airway pressure * FiO_2_ * 100]/PaO_2_) the PaO_2_ needs to be determined in arterial blood samples. The SpO_2_/FiO_2_ ratio can be calculated if SpO_2_ < 98%. If no indwelling arterial line is present, the OSI  ([mean airway pressure * FiO_2_ * 100]/SpO_2_) is used. Haemodynamic parameters are read from the patient monitor; cumulative fluid balance is calculated as the sum of daily fluid balance; it is normalised to actual bodyweight. Calculation of the non-respiratory PELOD-2 requires the Glasgow Coma Score, pupillary reaction to light (both reactive/both dilated), lactate, mean blood pressure, creatinine, white blood cell count, and platelets. Blood sampling will be done using the indwelling arterial line or central venous line. The pRIFLE criteria require assessment of serum creatinine and urine output as described elsewhere [28].

Published criteria are used to study the prevalence of critical illness polyneuropathy and myopathy (CIPNM) [19, 31]. The diagnosis CIPNM is present if the patient has possible limb weakness (i.e. inability to raise the limbs against gravity upon a stimulus), electrophysiological evidence of axonal motor and sensor polyneuropathy, and if the patient is difficult to wean from the ventilator and no apparent cause is present. Strength of muscle limbs will be assessed daily by the attending physician as part of the routine clinical examination after 48 h after randomisation until the moment of discharge from the PICU. If this is present and the patient cannot be weaned of the ventilator, a paediatric neurologist is asked for consultation and electromyographic investigations are indicated. The latter two are part of routine care in patients with unexpected muscular weakness. Only then the diagnosis of CIPNM is confirmed or refuted. Centre for Disease Control definitions for ventilator-associated pneumonia (VAP) will be used [32]. Both CIPNM and VAP will be assessed daily by the attending physician as part of the routine clinical examination 48 h after randomisation until the moment of discharge from the PICU.

Assessment of the level and time course of the cumulative respiratory morbidity score assessed at baseline, 3, 6 and 9 months after PICU discharge will be done using the same questionnaire as described above. LCI and FRC will be measured 12 months after PICU discharge using multiple breath washout techniques [33]. Such lung function testing is feasible and validated, even in small children. Testing will be performed by dedicated paediatric pulmonology function testing technicians in participating centres that are equipped with this technique.

#### Exploratory outcome measures

Prevalence of drug withdrawal symptoms and delirium will be assessed according to local scoring system. Assessments will be done daily. Ventilator-free days at day 28 (VFD) is defined as the number of days within 28 days that a subject is alive and free of MV [34]. Patients will be assigned 0 VFD if they remained intubated or died prior to day 28 without remaining extubated for more than 24 h, or if they were cannulated for ECLS. Mortality is defined as PICU mortality and mortality 90 days after PICU discharge. The Pediatric Cerebral Performance Category and Pediatric Outcome Performance Category are calculated as described elsewhere [35].

All handling of personal data will be done according to the European General Data Protection Regulation. All data and samples will be collected and stored under a pseudonym. The subject’s identification code list will be available to the project leader and the investigator. Data will be collected by the research nurses and/or site-investigators. Study data were collected and managed using Research Electronis Data Capture (REDCap) electronic data capture tools hosted at UMCG [36, 37]. Data will be stored for 15 years. Samples will be stored at the University Medical Centre Groningen.

### Plans to promote participant retention and complete follow-up {18b}

Upon discharge, parents or legal caretakers are asked for their e-mail address and phone number; the personalised link to the web-based questionnaire will be e-mailed to them 3, 6, 9 and 12 months after PICU discharge. If there is no response within 7 days, they will receive a reminder via e-mail to promote participant retention.

### Data management {19}

Data are collected through case report forms in REDCap. Range checks for data values were added in the Code of Federal Regulations (CFR), if possible, to promote data quality. Data management is coordinated by the UMCG researchers and Informatie Management (IM) onderzoek UMCG. All participants receive a trial ID, and the personal details are known only to the researchers, pharmacy, and attending physician. Double data entry and validation are carried out by all participating centres. Monitoring of the data and trial proceedings is coordinated by the UMCG. Monitoring of the data and trial proceedings is carried out per research site before the start of the trial, soon after the start of the trial, and three times a year for the duration of the trial. A close-out visit is carried out after completion of the inclusion period per research site. Trial auditing is carried out only by invitation from the hospital board and its frequency is not specified.

The UMCG, as the coordinating centre, owns intellectual property. The investigators have unlimited access to the final dataset. A newsletter with the study results will be made available on the website of the patient and parent organisations and will be sent to participants upon request. Public access to the data and data sharing is in line with the guidelines of ZonMw (data management plan), the main funder of this trial. Data access is restricted to authorised use only, and access will be granted by the researchers upon reasonable request.

### Confidentiality {27}

Identifying information will be removed and substituted by an unique study number. Nonetheless, a randomisation list will be remained with the name linked to the study number. The randomisation list will be securely stored by the local investigator and is only available for the study team and on request for the monitor, data safety monitoring board and the pharmacy. After the trial, the data will be stored secured at the secured long storage of the UMCG until it is deemed appropriate to be destroyed.

### Plans for collection, laboratory evaluation, and storage of biological specimens for genetic or molecular analysis in this trial {33}

The pulmonary inflammatory response is measured in non-bronchoscopic wedged broncho-alveolar lavage (BAL) fluids and the systemic inflammatory response in plasma samples [38]. Measurement of pro-inflammatory cytokines and chemokines will be done using Luminex bead technology. Blood samples and BAL fluid are pre-processed and stored per specific standard operating procedures at the participating sites. Full analysis of the samples will be coordinated by the UMCG.

## Statistical methods

### Statistical methods for primary and secondary outcomes {20a}

Data is analysed with an intention-to-treat approach. The primary outcome measure is a continuous outcome parameter. First, it will be tested for normality using the Shapiro-Wilk test. Dependent upon the outcome of this test, in the univariate analysis, the primary outcome will be displayed as mean ± standard deviation or median (25–75 interquartile range) and analysed using either the Student *t* test or the Mann-Whitney *U* test. Paired analysis will be used to compare the primary outcome with its baseline value. In multivariate analysis, zero inflated regression analysis is used to adjust for possible confounding by age, gestational age, family history of asthma and/or allergy, season in which questionnaire was filled out, parental smoking, and parental perception of the outcome of randomisation. Depending on the outcome of the analysis on randomisation outcome, variables that were significantly different between the intervention and control arm will also be adjusted for in the multivariate analysis. On top of that, we will adjust if there is an imbalance in the use of (non-) pulmonary specific ancillary treatment used during PICU admission.

#### Secondary outcomes

For the secondary outcome measures, repeated measures will be tested using generalised estimating equations (GEE), adjusting for the outcome of randomisation and time and any other confounding factors applicable to the outcome measure. Non-repeated continuous measures will be compared between using either the Student *t* test or the Mann-Whitney *U* test, non-repeated dichotomous variables will be tested using the Chi-squared test or the Fisher Exact test if the absolute value is below 5. They will be displayed as a percentage (%) of the total with a 95% confidence interval.

### Interim analyses {21b}

The data safety monitoring board (DSMB) will monitor the study for efficacy and adverse events. Two interim analyses are planned after the inclusion of 50 and 100 patients respectively. For safety reasons, the study will be terminated prematurely if there is a significant increase in critical illness polyneuropathy and myopathy in these interim analyses (i.e. a threefold increase in prevalence) or for futility if there is no difference in the primary outcome measure.

### Methods for additional analyses (e.g.) subgroup analyses) {20b}

There will be no subgroup analyses performed.

### Methods in analysis to handle protocol non-adherence and any statistical methods to handle missing data {20c}

All study participants who have received a dose of the study drug will be included in a modified intention to treat analysis. Participants who have received the study drug during 48 h and with a respiratory morbidity score 12 months after discharge (primary outcome measure) will be included in the per-protocol population.

### Plans to give access to the full protocol, participant level-data and statistical code {31c}

The datasets analysed during the current study are available from the corresponding author upon reasonable request after obtaining approval of the use by all members of the investigative team.

## Oversight and monitoring

## Composition of the coordinating centre and trial steering committee {5d}

The trial steering committee is composed of the principal investigator (PI) and eight co-investigators, who all contributed to and approved the final protocol. The PI is responsible for oversight of the entire study. The co-investigators collaborated to the drafting of the study protocol, including the pulmonary function testing done at follow-up and the neurological muscle strength assessment during PICU admission. One co-investigator oversees the production and handling of the study medication, another one contributes to the implementation strategy and one co-investigator is responsible for the health care technology assessment. There is one research nurse on the steering committee and one biostatistician. Each participating site has a local PI who is responsible for the conduct of the study at their site and a research nurse to assist.

### Composition of the data monitoring committee, its role and reporting structure {21a}

There is a Data Safety and Monitoring Board (DSMB) for this study, composed of one paediatric intensivist who chairs the DSMB, two adult-based intensivists and one biostatistician/epidemiologist. Roles and responsibilities of the DSMB are detailed in a DSMB document. The DSMB meets at least once a year; the exact frequency of meetings will depend on trial events. Two interim analyses by the DSMB are planned after 50 and 100 inclusions.

### Adverse event reporting and harms {22}

For this study, the following adverse events (AE) are defined pneumothorax, decubitus, CIPNM, VAP, withdrawal syndrome, delirium, hypotension or tachycardia in response to study medication with need for intervention by means of medication or fluid challenge, allergic reaction and reintubation. These AEs will be recorded in the database. A serious adverse event (SAE) is any untoward medical occurrence or effect that cannot be attributed to the underlying disease or that can be expected in the natural disease course of paediatric acute respiratory distress syndrome. For this study, the following SAEs are defined death (all-cause mortality), hypersensitivity or an allergic reaction that is not attributable to any concurrent medication, defined by skin rash and/or hypotension and/or severe bronchospasm necessitating the use anti-histaminic drugs and corticosteroids and/or vasopressors, cardiac arrhythmias or dysrhythmias and clinically relevant incidents, judged by the investigator, which are unexpected within the natural course of PARDS. An elective hospital admission will not be considered as a serious adverse event.

The risks associated with this study are minimal based on the following arguments

- Patients in the intensive care unit are under constant tight observation, so any change in vital parameters is noted immediately. Furthermore, patients with severe lung injury are commonly deeply sedated
- Blood samples are only taken from an indwelling arterial catheter or central venous catheter, which are already in place for clinical purposes. Blood samples for this study will be combined as much as possible with routine blood sampling part of daily clinical care
- Endotracheal suctioning is routinely performed in mechanically ventilated patients by nurses taking care of the patients; for this study suctioning specimens are collected to measure the pulmonary inflammatory response
- The investigational drug is commonly used in (paediatric) critical care; hence, there is a good understanding of this drug.

### Frequency and plans for auditing trial conduct {23}

For this trial, we plan approximately 3 monitor visits per year per outlined monitoring plan.

### Plans for communicating important protocol amendments to relevant parties (e.g. trial participants, ethical committees) {25}

Protocol amendments will be submitted to the appropriate ethics committee. All agreed protocol amendments will be clearly recorded on a protocol amendment form and will be signed and dated by the original protocol approving signatories. All protocol amendments will be submitted to the relevant institutional IRB for approval before implementation, as required by local regulations. The only exception will be when the amendment is necessary to eliminate an immediate hazard to the trial participants. In this case, the necessary action will be taken first, with the relevant protocol amendment following shortly thereafter. In case of amendments that will have a direct impact on the participants, this will be communicated with the legal caretakers/participants by the contact information which is collected by enrolment.

### Dissemination plans {31a}

Individual-level de-identified patient data will be made publicly available after the study-specific aims have been published. The statistical analyses will be available for those who request them based on published analyses. Authorship of the final report will be based on contribution to the trial as determined by the principal investigators. The final report will be published in a peer-reviewed journal to facilitate communication to healthcare professionals and the public. Published results will be shared with study participants should they indicate an interest in receiving this information (e.g. publications of these data will be sent as a pdf to their email address).

## Discussion

This is the first paediatric trial evaluating the effects of muscular paralysis in moderate-to-severe PARDS. The proposed study addresses a huge research gap identified by PALICC by evaluating a practical need regarding the treatment of PARDS [39]. Paediatric critical care practitioners are inclined to use interventions such as NMBAs in the most critically ill. Data from the Paediatric ARDS International Epidemiology (PARDIE) study showed that approximately one of every three mechanically ventilated PICU patients received continuous NMBAs [13]. The use of NMBAs increased with PARDS severity and was more common among patients who also received inhaled nitric oxide (iNO) and HFOV. However, this liberal use of NMBAs must be weighed against potential side-effects, with CIPNM being the most prominent, a phenomenon that has been observed especially in adults who are on concurrent corticosteroids or have renal failure [19, 20]. The proposed study will provide much needed scientific support in the decision-making to start NMBAs in moderate-to-severe PARDS.

The primary outcome of this proposed study is a functional one, measured during follow-up. We choose this outcome for two reasons. First, based on the relatively low but highly variable PARDS mortality rates, the proposed study would require a large sample size. Consequently, such a study would have to run for many years, thereby potentially making the study not only not feasible but also creating the risk of the research question becoming less important [1]. Second, the focus in paediatric critical care research is redirecting towards functional outcomes [40, 41]. We think that this makes our primary outcome measure highly relevant. The maximum level of pulmonary function reached during childhood is a crucial determinant for respiratory function in adults [42–45]. Any event that causes lung injury and thereby reduces the level of pulmonary function may thus exert a negative impact on later life. This may especially be true for lung injurious events during early childhood (i.e., children < 8 years of age) when the lung is still developing. MV needs to be considered such an event in our perspective, which makes pulmonary function an important outcome measure.

The major threat for this trial is subject enrolment, especially in the context of COVID-19 disease. Many PICUs have seen a strong decline in acute respiratory illnesses due to the various preventive measures such as social distancing, school closures and lockdowns [46, 47]. However, we expect that with the progress that is being made with COVID-19 immunisation, PICU admission characteristics will return to the situation before the COVID-19 pandemic.

NMBAs have the potential to become a cheap therapeutic intervention for paediatric patients with moderate-to-severe ARDS. If shown to be effective, the results of this trial will be implemented in international guidelines and may pave the way for a better and personalised treatment.

### Trial status

The trial is, at the moment of writing, enrolling participants since the 1st of May 2019 and using protocol version 7 (March 1, 2019) is used. The trial has started in UMCG and will soon start in the other centres. The COVID-19 pandemic has been delaying the start of the other centres but also the inclusion rate has been lower than expected. Enrolment is expected to take 3 additional years and is expected to be fulfilled by August 2024

## Abbreviations

AE: Adverse event
ANI: Analgesia nociception index
ARDS: Acute respiratory distress syndrome
BAL: Bronchoalveolar lavage
CFR: Code of Federal Registration
CIPNM: Critical illness polyneuropathy and myopathy
DSMB: Data safety monitoring board
ECLS: Extracorporeal life support
ERT: Extubation readiness test
FRC: Functional residual capacity
GEE: Generalised estimating equation
HFOV: High-frequency oscillatory ventilation
iNO: Inhaled nitric oxygen
IRB: Institutional review board
LCI: Lung clearance index
MV: Mechanical ventilation
NMBA: Neuromuscular blocking agent
OI: Oxygenation index
OSI: Oxygenation saturation index
PALICC: Paediatric Acute Lung Injury Consensus Conference
PEMVECC: Paediatric European Mechanical Ventilation Consensus Conference
PARDIE: Paediatric ARDS International Epidemiology
PARDS: Paediatric acute respiratory distress syndrome
PEEP: Positive end-expiratory pressure
PELOD-2: Non-respiratory pediatric logistic organ dysfunction 2 score
PI: Principal investigator
PIP: Peak inspiratory pressure
PICU: Paediatric intensive care unit
pRIFLE: pediatric Risk, Injury, Failure, End-stage renal failure score
RCT: Randomised controlled trial
REDCap: Research Electronic Data Capture
ROSE: trial Re-evaluation Of Systemic Early Neuromuscular Blockade
SAE: Serious adverse event
VAP: Ventilator-associated pneumonia
VFD: Ventilator free days
VILI: Ventilator-induced lung injury
